# Allograft tendon reconstruction of the anterior talofibular ligament and calcaneofibular Ligament in the treatment of chronic ankle instability

**DOI:** 10.1186/s12891-017-1492-6

**Published:** 2017-04-08

**Authors:** Weikai Wang, Guo Hong Xu

**Affiliations:** Department of Joint and Sports Medicine, Affiliated Dongyang Hospital of Wenzhou Medical University, No 60, Wuning Xi Road, Dongyang, Zhejiang People’s Republic of China

**Keywords:** Lateral instability of the ankle, Ultrasound, Anatomical reconstruction, ATFL, CFL

## Abstract

**Background:**

The purpose was retrospectively to investigate functional and clinical outcomes after anterior talofibular ligament (ATFL) and calcaneofibular ligament (CFL) reconstruction using a single allograft.

**Methods:**

Patients with severe chronic lateral instability of the ankle underwent surgery after conservative treatment failed. Ultrasounds of the ankle were performed, and if the AFTL and CFL were completely torn without enough soft tissue for repair, the ligaments were reconstructed using allograft tendon. Outcomes were assessed by clinical examination, stress radiography, ultrasound, the American Orthopaedic Foot and Ankle Society score (AOFAS), and Karlsson Ankle Functional score (KAFS) before surgery and at final follow-up.

**Results:**

Nineteen patients, ten men and nine women with mean age of 27.9 years (range, 19–41 years), underwent reconstruction. Mean follow-up was 30 months (range, 24–40 months). At final follow-up, all patients had returned to activity without instability, pain, or limited range of motion. On stress radiography, mean talar tilt angle decreased from 17.32° ± 3.58° before surgery to 4.16° ± 1.12° at follow-up (*p* < 0.001). Mean anterior drawer test (ADT) distance decreased from 9.79 ± 1.01 mm before surgery to 3.97 ± 0.99 mm at follow-up (*p* < 0.05). Mean AOFAS improved from 64.00 ± 18.43 to 90.32 ± 5.17 points (*p* < 0.001), and mean KAFS improved from 50.84 ± 16.73 to 90.89 ± 5.08 points (*p* < 0.001). Ultrasound showed the reconstructed ligaments maintained good continuity and excellent tension. No case of infection and immunological rejection was reported.

**Conclusion:**

This novel reconstruction technique takes into account the anatomical specialty of AFTL and CFL. This case series showed increased stability of the ankle in clinical and functional outcomes.

**Trial registration:**

The trial registration number (TRN) and date of registration: ChiCTR-ORC-17010796, Mar 6th 2017. Retrospectively registered.

## Background

Ankle sprain is one of the most frequent sports injuries, which often results in ligament rupture, mainly the anterior talofibular ligament (ATFL) and calcaneofibular ligament (CFL) [1]. Because of poor healing, the injured ankle will gradually develop ankle instability, which is potentially devastating [2].

The collateral ligaments of ankle consist of three main ligaments, including the ATFL, CFL, and posterior talofibular ligament (PTFL) [3, 4]. The PTFL originates from the posteromedial surface of the fibula, stretching transversely and medially to insert broadly on the nonarticular posterior surface of the talus [5]. The ATFL, which originates from the fibular tip and courses to the talus neck, is an important stabilizer of the ankle, serving as the important restraint to ankle inversion. The CFL, which originates just below the AFL origin site and attaches to the lateral aspect of the calcaneus, provides static stability to the ankle and also safeguards the subtalar joint [6]. It has been reported that ATFL and CFL synergistically provide rotation and inversion constraints necessary for ankle stability [4].

Anatomic reconstructions aim to recreate the disrupted ATFL and CFL regarding the anatomic factors. Surgery mainly utilizes a tunnel through the fibular tip and talus neck to facilitate graft passage and reconstruction of both ATFL and CFL [7–11]. We developed a simple reconstructive technique, which does not require the use of an interference screw in the talus neck. Our technique uses one allograft tendon to reconstruct both ATFL and CFL.

In addition, there is a debate about which patients need both both ATFL and CFL reconstruction. Traditional stress X-ray image of the talocrural joint provides evidence of the lateral ankle stability; however, it may not directly describe the condition of the ligaments. If the osseous architecture is abnormal or dynamic stabilizers are injured, stress radiography results will be positive even though the ligaments are normal [3, 4]. Therefore, it is necessary to use a diagnostic tool to directly evaluate the ligaments of the ankle.

Therefore, the aim of this study was to investigate the clinical and functional results in patients who underwent this novel technique of ATFL and CFL reconstruction and to also investigate the use of ultrasound to assess the ATFL and CFL.

## Methods

### Patients

This study was approved by the ethics committee of our hospital, and all subjects gave their informed consent. Patients with symptomatic unilateral chronic ankle instability between Aug 2012 and Jan 2014 decided to undergo surgery after long-term conservative treatment failed. They were carefully examined clinically and with stress radiography. The American Orthopaedic Foot and Ankle Society score (AOFAS) and Karlsson Ankle Functional score (KAFS) were used to assess the function of the ankle before the operation and at follow-up.

### Decision regarding the ligaments for reconstruction

Before surgery, standard B-ultrasound of the ankle was performed to decide whether to perform reconstruction and, if so, which ligament to reconstruct. If the ATFL and CFL were completely torn and soft tissues were insufficient for repair, we decided to reconstruct both ATFL and CFL.

### Surgical techniques

One senior surgeon experienced in this technique performed all of the reconstructions. The patient was positioned supine with a leg supporter holding the ipsilateral leg under general anesthesia. Arthroscopy was carried out on all patients, the anterior talofibular and calcaneofibular ligaments were evaluated, and any minor accompanying injuries were treated simultaneously.

**Fig. 1:**
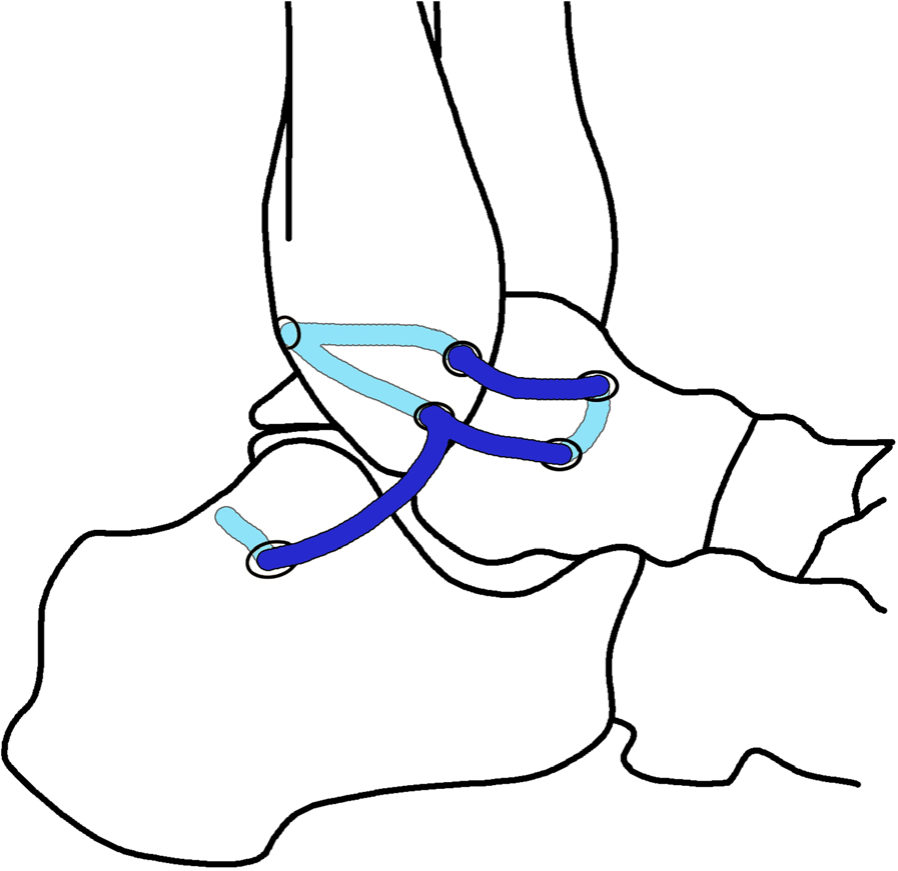
Surgcial schematic diagram

After arthroscopy, an oblique incision, about 5 cm in length, was made from the fibular tip, ending at the talar neck. The proximal edge of inferior extensor retinaculum was mobilized and tagged for later use. The conjoined ligament insertion of the ATFL and CFL at the malleolus was exposed after incision of the capsular. The ATFL insertion at the talus neck was also exposed. Two oblique 3.5-mm tunnels were created to make a “V” shape bone tunnel at the ATFL and CFL fibular insertion site. Then, another two 3.5-mm converging tunnels were made at the ATFL talus insertion site. A third 6-mm bone tunnel was made at the CFL calcaneus insertion site (Fig. 1).

A fresh-frozen tendon allograft (semitendinosus tendon) was prepared. After soaking in sterile saline for 20 min at room temperature, the tendon was trimmed and rolled to approximately 3.5 mm in diameter and 20 cm in length. Each end of the graft was sewn using nonabsorbable #2 braided polyester sutures. After pretension for 15 min, the allograft was pulled into the calcaneus bone tunnel and fixed with a 7 mm milagro screw (DePuy Mitek, USA). The other end of the graft was routed beneath the peroneal tendons, and pulled into the inferior fibular tunnel. The graft was then routed back through the superior fibular tunnel, and passed though the two tunnels in the talus, and sutured to the tendon at the fibular insertion site in a neutral position. The capsule and inferior extensor retinaculum was sutured back to cover the graft, and the incision was closed.

### Rehabilitation

All patients completed a same rehabilitation protocol, with the patient kept in the splint and nonweightbearing for 2 weeks and using crutches and the rehabilitation brace for next 6 weeks postoperatively. Full weightbearing was allowed at 2 to 3 weeks after surgery, and full range-of-motion exercises and triceps surae muscle were allowed accordingly. Patients were suggested to return to sports at 8 to 12 months, which is depended on their progress with physiotherapy.

### Clinical assessments and follow-up

One physician, which was not involved in the patients’ rehabilitation, completed all clinical assessments. Ankle range of motion (ROM) was measured using a goniometer. Stress radiography was performed to assess laxity of the ankle. AOFAS and KAFS were used to assess functional outcomes. We performed ultrasound examination to evaluate the condition of the reconstructed graft after surgery.

### Statistical methods

Statistical comparison was performed with STATA 10.0 software (Stata Corp, USA). The data was reported as mean ± standard deviation (SD). The paired Student *t-*test was used to compare the continuous variables between the groups. Statistical significance level was set at 0.05.

## Results

The results of standard B-ultrasound identified 19 patients with complete tears of the ATFL and CFL, and during the operation, these results were confirmed by arthroscopy. The 19 patients (ten men and nine women) had mean age at operation of 27.9 years (range, 19–41 years). The left ankle was injured in eight cases, and the right, in 11 cases. The interval between the initial ankle sprain and the surgery time ranged from 12 months to 10 years (average, 2.4 years). All patients had completed long-term conservative treatment but still had recurrent ankle sprains with severe lateral ankle instability.

Mean follow-up time was 27 months (range, 24–30 months). There were no cases of postoperative infection or immunological rejection, and none of the patients had an ankle sprain during follow-up.

Ankle instability in all patients resolved. No patients had limited ROM. At the follow-up time, there was no significant difference of ROM between the injured and contralateral ankle (Table 1).

**Table 1:** Range of motion

Motion	Reconstructed ankle	Normal ankle
Dorsiflexion	24.2° ± 5.84°	24.7° ± 4.85°*
Plantarflexion	43.7° ± 5.74°	43.4° ± 4.10°*

*P < 0.05 compared with the value before surgery

Stress radiography showed that the ankles of all patients were stable in the anterior drawer test (ADT) and inversion test. Mean talar tilt angle measured manually was 17.32° ± 3.58° (range, 13–25°) before surgery and 4.16° ± 1.12° (range, 3–7°) at follow-up (*p* < 0.001). Mean anterior drawer test distance decreased significantly from 9.79 ± 1.01 mm (range, 8–11 mm) before surgery to 3.97 ± 0.99 mm (range, 3–6 mm) at follow-up (*p* < 0.001).

Mean AOFAS improved significantly from 64.00 ± 18.43 points (range, 31–84 points) before surgery to 90.32 ± 5.17 points (range, 84–100 points) at follow-up (*p* < 0.001). Mean KAFS also improved significantly from 50.84 ± 16.73 points (range, 20–75 points) before surgery to 90.89 ± 5.08 (range, 85–100) at follow up (*p* < 0.001).

### Ultrasound

Ultrasound showed that the reconstructed anterior talofibular ligament spanned across the anterolateral ankle joint from the fibular tip to the talus neck. The reconstructed calcaneofibular ligament originated below the ATFL origin site, coursed deep to the peroneal tendons, and inserted to the lateral aspect of the calcaneus. Both of the ligaments showed good continuity via ultrasound. With extension and flexion, the two reconstructed ligaments maintained excellent tension.

## Discussion

Numerous procedures for ATFL and CFL reconstruction have been reported with various successful rate. Sugimoto and colleagues [10] used a bone-patellar tendon graft to reconstruct the ATFL and CFL in 13 patients with chronic ankle instability. At mean follow-up of 26.5 months, the average talar tilt degree improved from 18.4° ± 5.5° preoperatively to 4.9° ± 2.6° postoperatively, and the average ADT decreased from 9.1 ± 2.6 mm preoperatively to 5.8 ± 1.6 mm postoperatively. According to the score devised by Good, six patients had a Grade III clinical condition and seven had a Grade IV condition preoperatively. After surgery, all patients had a grade I condition.

Coughlin and colleagues [11] reported another surgical technique using a free gracilis tendon transfer to anatomically reconstruct the ATFL and CFL. At a mean follow-up of 23 months, the outcomes of 24 of 28 patients were regarded as excellent and four of 28 as good, according to subjective self-assessment, pain scores, AOFAS, and KAFS. Talar tilt degree decreased from 13° preoperatively to 3° postoperatively, and the ADT decreased from 10 mm preoperatively to 5 mm postoperatively.

Pagenstert and colleagues [12] described their technique to anatomically reconstruct ATFL and CFL using a free plantaris tendon graft without screw fixation. At a mean follow-up of 3.5 years in 50 patients, the mean AOFAS was 97.9. In 39 cases (78%), the outcome was graded as excellent, and in 9 cases (18%), as good. The reconstruction addressed both ATFL and CFL in a double fashion with great attention to normal kinematics of the ankle.

In the present study, we report data consistent with the previously described literature. The functional scores improved significantly after compared with before surgery. The reconstruction technique used was similar to that described by Pagenstert and colleagues [12], in that the anterior talofibular ligament was reconstructed in a double fashion that respected the normal kinematics of anterior talofibular ligament. However, our technique is unique in that the calcaneofibular ligament was reconstructed with a single tendon graft with an interference screw to fix the insertion. We believe that this is a simpler and safer technique to firmly fix the tendon end, compared with the procedure of Pagenstert and colleagues. The single reconstructed calcaneofibular ligament is much closer to the native calcaneofibular ligament.

Furthermore, unlike Coughlin and colleagues [11] who performed single tendon reconstruction of the anterior talofibular ligament with interference screw fixation on the talar insertion, for several reasons, we prefer to create a bone bridge on the talar neck and reconstruct the anterior talofibular ligament in a double fashion. First, it is much closer to the native anterior talofibular ligament. Second, it is much easier to drill a 3.5-mm diameter tunnel than a 6-mm or 7-mm diameter tunnel on the small fibular tip and maintain a strong bone bridge. Our reconstructive technique respects the anatomical feature of ATFL and CFL. This orientation and attachment of the reconstructed ATFL and CFL are similar to those described by Burks and colleagues [9]. With the use of these specific anatomical data, the anatomical sites for ligament attachment can be located more precisely during reconstruction. The main disadvantage of this surgical technique is lack of a biomechanical cadaveric study to verify the benefits of our technique.

With regard to allograft problems, no infections or immunological rejection occurred in our study. Allograft tendons have been widely used for reconstructive surgery, especially in the knee joints, and no infections or immunological rejection have been reported [13–15]. Caprio and colleagues [2] described an augmented reconstructive technique of the ATFL and CFL with a semitendinosus tendon allograft, and they advocated this procedure as a safe, effective method to manage lateral ankle instability.

To decide the surgical treatment, increasingly accurate diagnostic tests are necessary. Lateral ligament rupture of the ankle has ever been diagnosed using stress x-ray image of the talocrural joint. Takao and colleagues [16] performed stress x-ray image of the subtalar joint to decide which ligaments needed reconstruction. In 17 patients, with the talocalcaneal angle less than 10°, only the ATFL was reconstructed; in 4 patients, with the talocalcaneal angle of 10° or more, both ATFL and CFL were reconstructed. After surgery, mean talar tilt angle on stress x-ray image of the talocrural joint decreased significantly in both groups. However, this approach provides only indirect evidence of the lateral stability of the ankle, which may not directly describe the condition of the ligaments. As described before, if osseous architecture is abnormal or dynamic stabilizers are injured, stress radiography results will be positive even though the ligaments are normal. Therefore, it is necessary to use a diagnostic tool to directly assess the ATFL and CFL.

In 2008, Takao and colleagues [17] used magnetic resonance imaging (MRI) to evaluate morphological changes of the ATFL and CFL, demonstrating its usefulness in evaluating the ligaments and directing treatment. The results of MRI are confirmed via arthroscopy, which allows direct visualization of the ATFL and CFL. In contrast, in this study, we used B-ultrasound to assess the ATFL and CFL, which helped us formulate a plan for surgery.

Although MRI has become the preferred tool for evaluating tendons and ligaments [18], B-ultrasound is a uniquely powerful diagnostic tool, with the ability to intensively examine an pathological area during motion [19–22]. The commonly stated shortcoming of so-called operator dependence, on the other hand, becomes a distinct advantage over any of the other radiology tests, particularly in evaluation of the ligaments. The ATFL and CFL have different anatomic characteristics in different people, including origin, insertion, and course direction. Because MRI is limited to three traditional views (axial, sagittal, and coronal), this may unavoidably lead to diagnostic errors because of the scanning slice [23]. In this respect, ultrasound can evaluate the anterior talofibular and calcaneofibular ligaments in any plane and any angle, and trace the ligaments along their entire course. In addition, ultrasound can target the specific site of the patient’s symptom(s) and dynamically observe the underlying structures during motion [24]. Such dynamic evaluation is very useful for the anterior talofibular and calcaneofibular ligaments, because both ligaments have different levels of tension in different people. Therefore, we believe dynamic ultrasound can provide important information for planning surgery. Fibers of the tendons and ligaments can be visualized clearly via ultraound, both before surgery to assess the extent of injury and after surgery to assess the condition of the reconstructed ligaments.

There were several limitations in this present study, including a relatively small sample size, lack of a stress-testing device, no control group, and anatomical reference. When both the ATFL and CFL are injured, we first consider direct repair. If there is not enough soft tissue for repair, we turn to reconstruction. According to the patients’ condition and willingness, the allograft tendon was preferred. Therefore, the number of patients who underwent reconstruction of the ATFL and CFL using allograft appears small. Additionally, we lack a stress-testing device to perform standard stress radiography. Instead, we performed standard stress radiography for the ADT and the inversion test manually. Finally, the anatomical data mentioned in our technique were based on adult patients with average body size. For others, the anatomical data should be adjusted correspondingly.

## Conclusions

We describe a novel technique which takes into account the anatomical specialty of anterior talofibular and calcaneofibular ligaments. Our series showed increased stability of the ankle in clinical and functional outcomes.

## Abbreviations

ADT: Anterior drawer test
AOFAS: American Orthopaedic Foot and Ankle Society
ATFL: Anterior talofibular ligament
CFL: Calcaneofibular ligament
